# Pica behaviors in schizophrenia: a call for further research

**DOI:** 10.1186/s40337-021-00472-y

**Published:** 2021-09-16

**Authors:** Mohsen Khosravi

**Affiliations:** Department of Psychiatry and Clinical Psychology, Baharan Psychiatric Hospital, Zahedan University of Medical Sciences, Zahedan, Postal Code: 9813913777 Iran

**Keywords:** Behavior, Pica, Schizophrenia

## Abstract

Pica as a scavenging behavior represents a serious health hazard to the care of patients with schizophrenia. Despite the rare comorbidity of pica eating disorder and schizophrenia, pica behaviors are relatively common in this group of patients (1.5% vs. 14.3%). The risk of multiple clinical problems such as electrolyte disturbances, intestinal obstruction, and heavy metal poisoning has made pica behaviors an important research topic in patients with schizophrenia. However, few studies have examined the etiology, mechanisms, and treatment of pica behaviors in schizophrenia. This letter is a call for further research into discovering the biopsychopathology of these major clinical manifestations among patients with schizophrenia.

## Main text

Despite the initial description of maladaptive eating behaviors among patients with schizophrenia by Eugen Bleuler in the early nineteenth century, they have remained poorly studied and understood in human eating research. These pathological behaviors, which occur at different stages of psychosis (i.e., premorbid, active, or residual phases), may complicate the diagnostic process by mimicking feeding and eating disorders. Recent studies have described various maladaptive eating behaviors among patients with schizophrenia. As one of the most important of them, pica behaviors are characterized by repetitive eating of nonnutritive and nonfood substances [1, 2]. In the context of schizophrenia, pica behaviors are relatively common, as Osuji and Onu reported an elevated rate of these incompatible behaviors (14.3%) in the early stages of schizophrenia by assessing 206 incident cases of schizophrenia [1]. However, underestimation and sometimes ignorance of these unusual behaviors among patients with schizophrenia may lead to clinical complications such as electrolyte disturbances, intestinal obstruction, and heavy metal poisoning [3].

Although pica behaviors have been previously reported in various forms such as saturnism (lead poisoning), coprophagia (ingestion of feces), potomania (drinking excessive amounts of beverages, around 8–10 L per day), or even deliberate foreign body ingestion among patients with schizophrenia, the etiologies of pica behaviors are still poorly known in this group of patients [2, 4]. According to the limited data in the area, some possible causes of pica behaviors in schizophrenia include: (1) Psychotropic-induced compulsive eating behavior of inedible materials, e.g., a case report of pica behaviors following continuous, chronic olanzapine treatment attributed to cortico-basal ganglia dysfunction via blocking the 5-HT2a receptors and increase of dopamine release in the mid-brain and frontal cortex; (2) Prolonged malnutrition or micronutrient deficiencies syndrome due to underlying symptoms of schizophrenia; (3) Comorbidity between obsessive–compulsive disorder and schizophrenia; (4) Hematopoietic inhibition induced by chronic schizophrenia or chronic use of psychotropic drugs; (5) Hyperorality in the context of cognitive deficits and temporal lesions; (6) As a manifestation of disorganization across the illness course of schizophrenia; (7) Secondary to delusional beliefs [3–5].

Therapy can include two separate approaches, namely pharmacological and cognitive-behavioral treatments, which seem to vary with patients’ characteristics and the specific behaviors involved [6]. Nevertheless, no clinical trials have yet been conducted to evaluate the effectiveness of pharmacological and cognitive-behavioral treatments among patients with schizophrenia suffering from pica behaviors; and the evidence available to health care decision-makers and clinicians is limited to few initial case reports [3–5].

Altogether, very little research has been devoted to discovering the biopsychopathology of pica among patients with schizophrenia. So, it is not unreasonable to expect that there will still be legitimate debates about the prevalence, etiology, and most effective treatments of pica behaviors in patients with schizophrenia. Further research is needed to clarify the etiology, mechanisms, and treatments of pica behaviors among patients with schizophrenia due to negative impact on physical functioning and serious medical consequences.

## Data Availability

N/A.
